# LncRNA NEAT1 Promotes Gastric Cancer Progression Through miR-17-5p/TGFβR2 Axis Up-Regulated Angiogenesis

**DOI:** 10.3389/fcell.2021.705697

**Published:** 2021-09-06

**Authors:** Yangwei Xu, Yanyan Li, Yue Qiu, Fei Sun, Guifang Zhu, Jingbo Sun, Guixing Cai, Wanmei Lin, Yun Fu, Hongmei Wu, Shanshan Jiang, Zhihui Wen, Feiyan Feng, Junjie Luo, Yuqin Yang, Qingling Zhang

**Affiliations:** ^1^Department of Pathology, School of Basic Medical Sciences, Southern Medical University, Guangzhou, China; ^2^Department of Pathology, Guangdong Provincial People’s Hospital, Guangdong Academy of Medical Sciences, Guangzhou, China; ^3^Nanfang Hospital, First Clinical Medical School, Southern Medical University, Guangzhou, China; ^4^Department of Pathology, Zhujiang Hospital, Southern Medical University, Guangzhou, China

**Keywords:** LncRNA NEAT1, miR-17-5p, gastric cancer, TGFβR2, angiogenesis, progression

## Abstract

**Background:**

Long non-coding RNAs (lncRNAs) have been indicated to play critical roles in gastric cancer (GC) tumorigenesis and progression. However, their roles in GC remain to be further elucidated.

**Methods:**

RT-qPCR and fluorescence *in situ* hybridzation (FISH) were conducted to detect the expression of lncRNA NEAT1 in GC tissues and cell lines. Gene Set Enrichment Analysis (GSEA) was performed to screen out potential phenotypes and pathways that NEAT1 may participate in. NEAT1-silenced AGS and MGC803 cells were constructed and a series of functional experiments to investigate the roles of NEAT1 in GC angiogenesis both *in vitro* and *in vivo*. RNA pull down and luciferase reporter assays were utilized to illustrate the mechanisms underlying the functions of NEAT1 in GC.

**Results:**

We observed that NEAT1 was upregulated in most GC specimens and cell lines. NEAT1 high was correlated with poor prognosis of GC patients. *In vitro* experiments showed that NEAT1 promoted GC angiogenesis by enhancing proliferation, migration, and tube formation ability of endothelial cells. Mechanism researches revealed that NEAT1 could competitively sponge miR-17-5p which targeted TGFβR2 directly. Subsequently, activate TGFβ/Smad pathway by following with upregulation of a series of classical proangiogenic factors especially VEGF.

**Conclusion:**

The study unveiled that the LncRNA NEAT1/miR-17-5p/TGFβR2 axis is a novel mechanism in GC angiogenesis. Disrupting this axis may be a potential strategy for GC treatment.

## Introduction

Gastric cancer is the fifth most frequent malignancies and the fourth leading cause of cancer death globally. In spite of the remarkable progression in diagnoses and therapies, overall prognosis of GC patients remains dismal (Siegel et al., 2021). Thus, developing effective targeted therapies for successful intervention is vitally important. Excessive angiogenesis is widely believed to fuel tumor proliferation and metastases, and is identified as a hallmark accounting for the poor prognosis of GC. Anti-angiogenic therapy has raised more and more interest due to its low response rate and the inevitable chemoresistance. However, the underlying molecular mechanisms that mediate GC angiogenesis have yet to be fully clarified.

Long-stranded non-coding RNAs are important members of the ncRNA family whose lengths exceed 200 nucleotides and have limited protein coding potential (Mercer et al., 2009). Growing evidences have indicated the critical roles that lncRNAs play in the tumorigenesis and progression of human cancers (Li et al., 2018). The lncRNA SATB2-AS1 suppresses colorectal cancer aggressiveness by repressing snail transcription and epithelial-mesenchymal transition (Wang et al., 2019). Dysregulation of lncRNAs result in aberrant expression of multiple genes through multiple mechanisms including the classical ceRNA mechanism. In this theory, lncRNAs decrease the expression of microRNAs (miRNAs) *via* sponging them, which lead to upregulation of miRNA targets. For instance, LncRNA LINC00662 promotes cell growth and metastasis by competitively binding with miR-340-5p to regulate CLDN8/IL22 co-expression and activating ERK signaling pathway in colorectal cancer (Cheng et al., 2020). LncRNA KCNQ1OT1 promotes cell proliferation and cisplatin resistance *via* sponging miR-211-5p to mediate the Ezrin/Fak/Src axis in tongue cancer (Zhang et al., 2018). Thus, it’s of great value to elucidate the mechanisms underlying lncRNA-mediated cancer progression and exploring more effective therapeutic targets for tumor patients.

Long-stranded non-coding RNAs nuclear-enriched abundant transcript 1 (NEAT1) is a pivotal component of nuclear paraspeckles, which have been reported to exert extensive roles in cancer progression (Clemson et al., 2009). Accumulating evidences have revealed that NEAT1 is dysregulated and acts as an unfavorable prognostic factor in human cancers including gliomas (Zhen et al., 2016), colorectal cancer (Wu et al., 2015), liver cancer (Wang et al., 2017), gallbladder cancer (Yang et al., 2020), and gastric cancer (Wang et al., 2018). NEAT1 promotes tumorigenesis and progression *via* enhancing malignant phenotypes of cancer cells including migration, invasion, proliferation, and chemoresistance. However, the correlation between NEAT1 and tumor angiogenesis has hardly been reported. MiR-17-5p was widely reported to function in progression of multiple cancers such as GC (Song et al., 2020), colorectal cancer (Xu et al., 2019) and nasopharyngeal carcinoma (Duan et al., 2019). Nevertheless, its regulatory mechanisms and connection with NEAT1 in GC remain to be further elucidated.

In this study, based on bioinformatics analysis and a series of *in vitro* and *in vivo* experiments, we first proved that NEAT1 correlated with enhanced angiogenesis in GC. Next, we demonstrated that NEAT1 competitively sponged miR-17-5p, which subsequently upregulated Transforming growth factor-β receptor 2 (TGFβR2) expression and activated the TGFβ/Smad pathway. These results deepen our understanding on GC angiogenesis and may provide feasible therapeutic strategies for GC patients.

## Materials and Methods

### Clinical Samples and Cell Culture

Sixty-four cases of gastric cancer and matched adjacent normal gastric tissue samples were obtained from patients undergoing surgical treatment at Nanfang Hospital, Southern Medical University. No chemotherapy or radiation therapy was administered prior surgery. Both study protocol and informed consent were approved by the Ethical Committee of Nanfang Hospital.

Human normal gastric mucosal cells GES-1, Human gastric cancer cell lines (AGS, MGC803, SGC7901, MKN45 and HGC27), and Human umbilical vein endothelial cell line (HUVEC) were obtained from the Cell Bank of Chinese Academy of Medical Sciences (Shanghai, China). These cells were cultured in PPMI-1640 medium supplemented with 10% fetal bovine serum (FBS, Thermo Scientific, Waltham, MA, United States), 100 IU/mL penicillin G and 100 μg/mL streptomycin (Invitrogen Life Technologies, Carlsbad, CA, United States). Cells were incubated in a humidified atmosphere containing 5% CO_2_ at 37°C. All cell lines were routinely tested for mycoplasma and the results were negative.

### RNA Isolation, Reverse Transcription, and Quantitative Real-Time PCR

Total RNA was extracted using Trizol (Invitrogen, United States). To quantify the expression of NEAT1 and proangiogenic factors, the total RNA was subjected to polyadenylation and reverse transcription (RT) using a ThermoScript^TM^ RT-PCR System (Invitrogen). Real-time polymerase chain reaction (PCR) analysis was performed using an SYBR Green PCR master mix (Applied Biosystems, United States) on an ABI 7500HT system. GAPDH was used as an endogenous control. All samples were normalized to internal controls, and fold changes were calculated through relative quantification (2^–ΔΔCT^). For miRNA detection, reverse transcription was performed and expression of miRNA was measured by All-in-One^TM^ miRNA qRT-PCR Detection Kit (GeneCopoeia) according to the use manual. Genomic DNA (gDNA) was isolated from tissues or cultured cells according to easy pure genomic DNA kit (Transgen Biotech). The primers used are shown in Supplementary Tables 2, 3.

### Western Blot

Cells were lysed in RIPA lysis buffer with protease inhibitor cocktail to extract total proteins. Before separating by SDS-PAGE gel and transferring onto the PVDF membrane, proteins were quantified by BCA protein assay kit (Pierce, KeyGEN BioTECH, China). Tris buffer containing 0.1% Tween-20 and 5% BSA was used to block the membrane at room temperature. Rabbit antibodies to GAPDH, VEGF (1:1000, Proteintech), and P-smad2, P-smad3, TGFβR2 (1:1000, CST) were used to incubate with the membrane overnight at 4°C, followed by HRP-conjugated secondary antibody treatment (anti-rabbit IgG/anti-mouse IgG, CST, 1:10000). After washed three times with PBST, the membrane was developed with ECL substrate and imaged using the enhanced chemiluminescence detection system (Tennon5200, China) as described by the manufacturer.

### Human Umbilical Vein Endothelial Cells (HUVEC) Tube Formation Assay

Growth factor reduced matrigel (BD Biosciences, United States) was prepared and kept on ice overnight until used. 50 μl matrigel was added to 96-well plate per well evenly and incubated at 37°C for at least 30 min. After digested with 0.25% trypsin and washed with PBS for three times, HUVEC cells (2 × 10^4^ per well) were resuspended with 200 μl serum-free medium and seeded onto the prepared matrigel. Images were photographed after incubation at 37°C for 4 h. The capillary tubes were quantified under a 100× bright-field microscope, by measuring the total numbers of the completed tube structure. Each experiment was repeated three times.

### Chicken Chorioallantoic Membrane (CAM) Assay

Chicken chorioallantoic membrane assay was performed as reported (Deng et al., 2019). Briefly, a window about 1.0 cm in diameter was opened in the eggshell to expose the CAM. A sterile rubber ring in 0.5 cm diameter was placed on the CAM and then 100 μl conditioned medium (CM) was added. The window was closed using a piece of steriled adhesive tape, and eggs were placed in a 37°C incubator with 80–90% relative humidity for 2–3 days. CAMs were fixed by stationary solution (methanol: acetone = 1:1) for 15 min before it was cut and harvested. Photos were taken by a digital camera (Cannon, Japan) and the effects of CM on angiogenesis were assessed through assessing the number of second- and third-order vessels.

### HUVEC Cell Proliferation Assay

Cell proliferation was analyzed with EdU assays according to the manufacturer’s instructions (KeyFluor488 Click-iT EdU Kit, keyGEN BioTECH, China). Cells cultured in 96-well plate were treated with 100 μL of medium containing 20 μM EdU and incubated at 37°C, 5% CO_2_ for 2 h. After washed with PBS for three times, the cells were fixed with 4% paraformaldehyde for 30 min and incubated with 0.5% Triton-X-100 in PBS for 20 min. The nuclei were stained with DAPI for 10 min. The images of five randomly selected areas of each group were taken with a fluorescence microscope (Leica, Germany) and the proliferation rate was calculated.

### HUVEC Cell Migration Assay

Transwell assay was performed to access migration ability of HUVEC. Firstly, 500 μl culture medium containing 20% FBS was added to the lower chamber and HUVEC cells (1 × 10^5^) were trypsinized and resuspended in 100 μl serum-free medium. Then the resuspended cells were seeded onto the upper chamber and incubated at 37°C for 24 h. Migrated cells attached to the bottom surface of the insert were then fixed with methanol and stained with hematoxylin. Penetrated cells were quantified under a light microscope in five random visual fields (200×).

Cell migration was also analyzed with wound healing assay. HUVECs were seeded to a 6 well plate and a 1000 μl plastic pipette tip was used to scratch the monolayers. After washed with PBS for three times, the wounded cells were then cultured in serum-free medium for an additional 12 h and photographed under a fluorescence microscope. The distance between the edge of the scratch wound was measured before and after cell migration. The mean migration distance (μm) was calculated by subtracting the length after 12 h from that at 0 h. The result was expressed as a migration index, i.e., the distance migrated by treated cells compared with the distance migrated by control cells.

### Enzyme-Linked Immunosorbent Assay (ELISA)

The supernatants from AGS and MGC803 were collected and the cell number was counted. The supernatant was used to measure the total levels of extracellular VEGF by the human VEGF ELISA Kit (Enzyme-linked Biotechnology, China) according to the manufacturer’s introductions. The cytokine expression level (pg/ml) per 1 × 10^5^ cells was analyzed.

### IHC, IF and FISH

Specimens of gastric cancer and matched adjacent normal gastric tissue samples were fixed with 4% formalin, paraffin embedded and sectioned (4 μm). The tissue sections were then deparaffinized and dehydrated followed by incubation in 3% hydrogen peroxide for 10 min. Slides were stained with primary antibodies against CD31 (1:100, ZSGB Bio) and Ki67 (1:100, ZSGB Bio) at 4°C overnight after blocking with 5% BSA in PBS for 1 h at RT. Corresponding secondary antibodies were used for 1 h at RT. Targeted molecules were detected following DAB staining for immunohistochemistry. Slides were finally counterstained with hematoxylin. Two independent investigators blinded to sample identify, one investigator performed the staining and another one analyzed the tissue section.

For immunofluorescent staining, tissue sections and cells were immunostained with primary antibodies against TGFβR2 (Proteintech, 1:200) and CD31 (1:100, Abcam) overnight at 4°C and subsequently incubated with fluorochrome conjugated antibodies. Finally, DAPI was added as nuclear counterstain. Images were captured using a fluorescence microscope or laser scanning confocal microscope (Nikon, Japan).

LncRNA NEAT1-FISH was conducted as previously described (Adriaens et al., 2016).

### Biotinylated RNA Pull-Down Assay

Pull down assay was carried out as described (Hu et al., 2019). In brief, 1 × 10^7^ cells were harvested, lysed and sonicated for detecting the LncRNA NEAT1 pull down miRNAs. The LncRNA NEAT1 probe was incubated with streptavidin magnetic beads (Beaver, china) for 2 h to generate probe-coated beads, then incubated with cell lysates. The products after incubation were then eluted with trizol, followed by qRT-PCR. For miR-17-5p pulled down, 1 × 10^7^ cells were collected, lysed, sonicated and incubated with streptavidin magnetic beads after transfected with biotinylated miR-17-5p mimics or mutant using lipofectamine2000 (Thermo Fisher, China), followed by washed, eluted and qRT-PCR.

### Dual-Luciferase Reporter System Analysis

For NEAT1 and miRNA luciferase assay, the NEAT1 sequences containing wild-type or mutated miRNA binding sites were, respectively, synthesized and inserted into pmirGLO luciferase vector (Jikai, China). MiR-17-5p mimics were then co-transfected with the above mentioned vectors into cells using lipofectamine2000. After 48 h transfection, cells were harvested, lysed, and subjected to luciferase activity detection by the Luc-pair^TM^ Duo-Luciferase HS assay kit (GeneCopoeia, China). Relative luciferase activity was normalized to the Renilla luciferase internal control. Three independent experiments were performed.

### Orthotopic Xenograft Tumor Mouse Model

The Orthotopic GC mouse models were constructed as described in our previous research (Li et al., 2020). Briefly, after anesthetizing the mice with ketamine (70 μg/kg), a small incision was made in the abdomen and to reveal the stomach. Then, 100 μl of a cell suspension (1 × 10^6^ cells) was injected into the muscle tissue of the stomach. Then, the stomach position was reset, the wound was treated with penicillin, and the abdominal incision was closed. Mice with digestive symptoms or moribund appearance were sacrificed. The tumor-bearing stomachs and livers were sectioned for immunohistochemical or immunofluorescence analysis.

### Statistical Analysis

Each experiment was performed at least three times. Statistical analyses were performed using Prism 7 (GraphPad Software, San Diego, CA, United States). Pearson’s chi-squared (χ2) test and student’s *t*-test were used to evaluate the significance of the differences among different groups. Survival curves were analyzed using the Kaplan-Meier method and assessed by log rank testing. All data were presented as the means ± standard deviation (SD).

## Results

### LncRNA NEAT1 Is Up-Regulated in Gastric Cancer, Predicts Poor Prognosis and Positively Correlates With Angiogenesis

**FIGURE 1:**
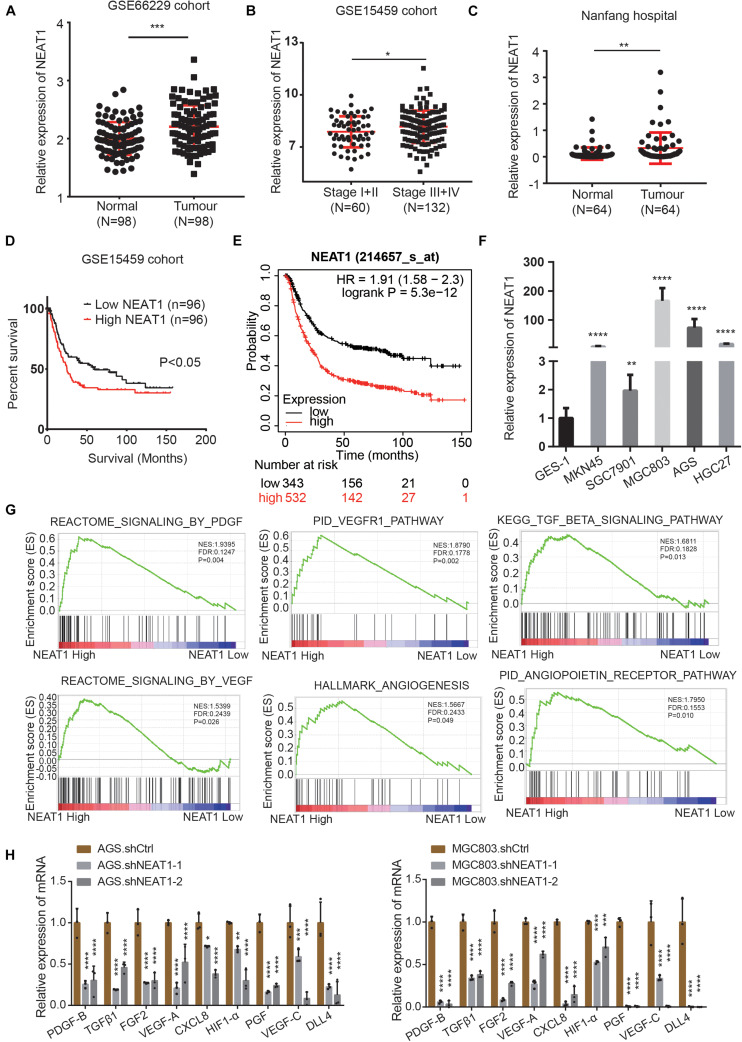
LncRNA NEAT1 upregulation correlates with poor prognosis and angiogenesis in GC. **(A)** Scatter diagram represents the expression level of LncRNA NEAT1 in GC (*n* = 98) and normal gastric tissues (*n* = 98) derived from the GEO GSE66229 dataset. **(B)** NEAT1 expression level in GC tissues with TNM stage I + II (*n* = 60) and stage III + IV (*n* = 132). Data were derived from GSE15459 dataset. **(C)** Expression levels of NEAT1 were examined by RT-qPCR in 64 GC tissues and their pair-matched adjacent normal tissues from Nanfang hospital. **(D)** Kaplan-Meier analysis was used to assess the relation between NEAT1 expression level and overall survival in GC patients from GSE15459 cohort. **(E)** Kaplan-Meier plotter analysis of the correlation of NEAT1 expression level with overall survival of GC patients by the KM Plotter database. **(F)** The expression levels of NEAT1 in the GC cell lines and normal stomach mucosal cell line were determined by RT-qPCR. **(G)** GSEA validated angiogenesis-related pathways in high NEAT1 expression GC cohorts of GSE15459 dataset. **(H)** RT-qPCR detects the effects of silencing NEAT1 on the expression of classical proangiogenic factors in AGS and MGC803 cells (mean ± SD, *n* = 3). *P < 0.05, **P < 0.01, ***P < 0.001, ****P < 0.0001.

To identify the roles and molecular mechanisms of LncRNA NEAT1 in gastric cancer (GC), we analyzed the expression of NEAT1 in GC across the GEO database. In GSE66229 cohort, we found that NEAT1 was significantly highly expressed in GC compared with normal gastric tissues (Figure 1A). In GSE15459 cohort, NEAT1 expression was higher in stage III and IV GC tissues than that in stage I and II (Figure 1B). Consistently, RT-qPCR results confirmed that NEAT1 expression was higher in 64 GC tissues than that in the matched adjacent normal mucosal tissues collected from patients undergoing surgery in Nanfang hospital (Figure 1C). The clinical and pathological characteristics of these patients are shown in Supplementary Table 1. Statistical analysis revealed that high NEAT1 expression levels were correlated with advanced T stage and big tumor size in GC patients (Supplementary Table 1). To explore the potential prognostic and predictive values of NEAT1 in GC progression, we next conducted survival analyses in the GSE15459 dataset and the Kaplan-Meier Plotter database^1^. The results consistently indicated that NEAT1 up-regulation was significantly correlated with shorter OS in GC patients (Figures 1D,E). We also detected NEAT1 expression through RT-qPCR in GC cell lines and GES-1, a normal stomach mucosal cell line, and observed that NEAT1 was higher expressed in GC cell lines compare with GES-1 cell line (Figure 1F).

To explore the potential regulatory role of NEAT1 in GC, the Gene Set Enrichment Analysis (GSEA) was performed in the GSE15459 dataset with 200 gastric cancer samples. As were shown in Figure 1G, pathways related to angiogenesis, including the VEGF, PDGF, TGFβ, and angiopoietin pathway, were positively enriched in patients harboring high NEAT1 expression. We next detected the mRNA expression of classical proangiogenic factors including TGFβ1, PDGF-B, VEGF-C, HIF-1α, PGF, FGF2, DLL4, VEGF-A, and CXCL8. The results showed that NEAT1 knockdown in AGS and MGC803 cells markedly decreased the expression of aforementioned proangiogenic factors (Figure 1H). Taken together, these findings suggested that NEAT1 might play an essential role in promoting GC progression *via* regulation of angiogenesis.

### Silencing NEAT1 Represses Tube Formation, Proliferation and Migration of Vascular Endothelial Cells

**FIGURE 2:**
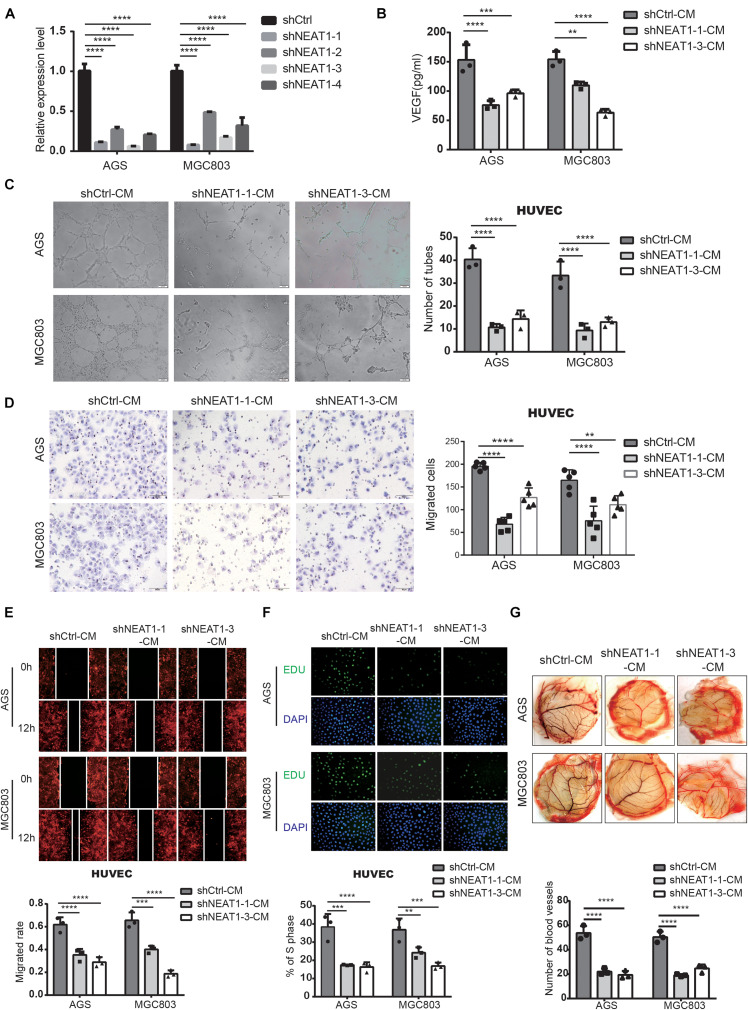
Silencing of NEAT1 suppressed angiogenesis of GC cells. **(A)** RT-qPCR assay was used to verify the successful construction of NEAT1 knockdown GC cells. **(B)** The concentration of VEGF was detected in the culture medium of NEAT1-2 silenced AGS and MGC803 cells by ELISA assay (mean ± SD, *n* = 3). **(C)** Representative capillary tubule structures were shown for HUVECs treated with culture medium collected from the NEAT1 silenced cells. Scale bar represents 50 μm. **(D,E)** Transwell **(D)** and wound healing **(E)** assay were performed in HUVECs to detect the effect of CM treatment on cell migration. Scale bar represents 50 μm. **(F)** EdU assay was performed in HUVECs to detect the effect of CM treatment on cell proliferation. **(G)** Representative images of blood vessels formed in the CAM assay after CM treatment. **P < 0.01, ***P < 0.001, ****P < 0.0001.

In order to investigate the biological behaviors of NEAT1 in GC cells, we firstly constructed two independent NEAT1 shRNA-expression lentivirus (shNEAT1-1 and shNEAT1-3). AGS and MGC803 cell lines with high endogenous NEAT1 expression were chosen to silence NEAT1. Transfection efficiency was confirmed by RT-qPCR (Figure 2A). To verify NEAT1’s potential roles in GC angiogenesis, we firstly detected the secretion of VEGF, a key secretory protein in regulating tumor vascularization, in the culture medium (CM) of GC cells with different NEAT1 expression levels through ELISA assays. The ELISA results indicated that silencing NEAT1 significantly decreased the secretion of VEGF in both AGS and MGC803 cells (Figure 2B). Tube formation assay was also performed and the results showed that NEAT1 silenced CM dramatically inhibited tube formation of HUVECs (Figure 2C). Besides, transwell assay (Figure 2D), wound healing assay (Figure 2E) and EdU assay (Figure 2F) were used to assess the effects of CM on HUVEC migration and proliferation, as the migration and proliferation of endothelial cells are critical for angiogenesis. The results showed that CM of NEAT1 silenced cells represented markedly weaker promoting effect on the migration and proliferation of HUVECs compare with the control group. The proangiogenic effect of NEAT1 was further verified *in vivo* by using the chick embryo chorioallantoic membrane (CAM) assay. As shown in Figure 2G, CM of NEAT1 silenced cells represented markedly weaker promoting effect on vessels formation of CAM (Figure 2G). Collectively, these findings revealed that silencing NEAT1 in GC cells significantly inhibited angiogenesis by disrupting tube formation of endothelial cells.

### LncRNA NEAT1 Upregulates TGFβR2 Expression by Sponging miR-17-5p as a ceRNA

**FIGURE 3:**
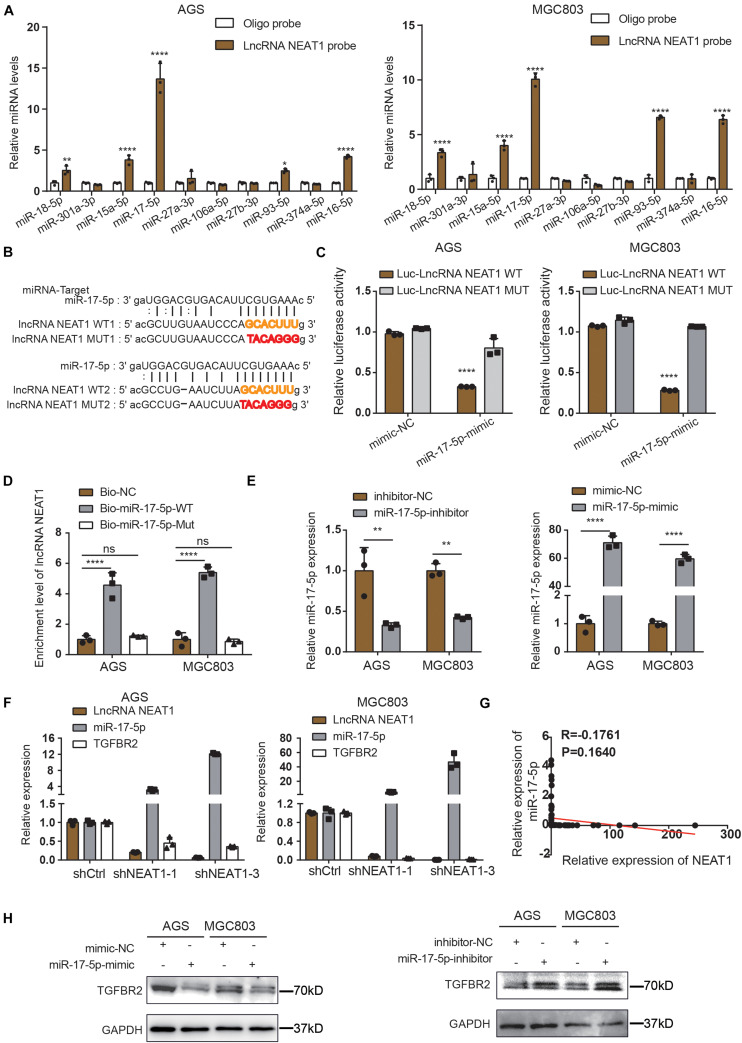
NEAT1 regulates TGFBR2 expression by directly targeting miR-17-5p. **(A)** The expression levels of 10 candidate miRNAs were detected by RT-qPCR after RNA pulldown assay. **(B)** Predicted binding sites of miR-17-5p on LncRNA NEAT1 are shown. **(C)** Luciferase activity was conducted in AGS and MGC803 cells co-transfected with luciferase reporter containing LncRNA NEAT1 sequences with wild type and mutant binding site of miR-17-5p and the mimic of miR-17-5p or control. **(D)** Biotin-coupled miR-17-5p wild type (Bio-miR-17-5p-WT) or its mutant (Bio-miR-17-5p-Mut) captured relative expressions of LncRNA NEAT1 in the complex. Relative level of LncRNA NEAT1 was normalized to input. **(E)** Transfection efficiency of miR-17-5p inhibitor (left panel) and mimics (right panel) in AGS and MGC803 cells were validated by RT-qPCR assay. **(F)** Relative mRNA levels of miR-17-5p and TGFβR2 detected by RT-qPCR after knockdown NEAT1 in AGS and MGC803 cells. **(G)** Regression analysis of GC tissue showed a negative correlation between miR-17-5p and NEAT1 (*n* = 64). **(H)** Effects of miR-17-5p inhibitor (above panel) and mimics (below panel) on TGFβR2 expression in AGS and MGC803 cells were validated by western blot. *P < 0.05, **P < 0.01, ****P < 0.0001.

Long non-coding RNAs most often function as competing endogenous RNAs (ceRNAs) to regulate downstream mRNAs by sponging miRNAs, suggesting that NEAT1 may exert its function in gastric cancer in a ceRNA manner. To elucidate the molecular mechanisms of NETA1 regulating angiogenesis in GC, bioinformatics analysis was conducted using starBase v2.0 to predict the miRNAs binding to NEAT1 and their potential binding sites. Ten miRNAs were identified as the potential targets of NEAT1 (Supplementary Table 4) and their interaction with NEAT1 was further verified through the RNA pulldown assay in both AGS (Figure 3A, left panel) and MGC803 cells (Figure 3A, right panel). What’s more, among the miRNAs detected, miR-17-5p appeared to be the most significant miRNA pulled down by the LncRNA NEAT1 probe. Thus, we postulated that NEAT1 might regulate angiogenesis mainly by sponging miR-17-5p as a ceRNA. To further validate the interaction between NEAT1 and miR-17-5p, NEAT1-wt and NEAT1-mut dual luciferase reporter vectors were constructed (Figure 3B) and co-transfected with miR-17-5p or miR-NC into GC cells. Luciferase reporter assay demonstrated that upregulation of miR-17-5p relatively reduced the luciferase activity of NEAT1-wt vector, but didn’t influence the luciferase activity of NEAT1-mut vector in AGS and MGC803 cell lines (Figure 3C). Additionally, RNA pull-down assay was implemented to determine whether miR-17-5p could directly bind to NEAT1. AGS and MGC803 cells were transfected with biotinylated miR-17-5p and then were harvested for biotin-based pull-down assays. As shown in Figure 3D by RT-qPCR, NEAT1 was pulled down by biotin-labeled miR-17-5p oligos but not the mutated oligos which disrupted base pairing between NEAT1 and miR-17-5p. Based on the above, our findings implied that miR-17-5p could directly bind to NEAT1.

To further elucidate the molecular mechanisms underlying the regulation of NEAT1/miR-17-5p axis, we examined miR-17-5p targets that were computationally predicted by the TargetScan algorithm. Among the high-scoring mRNA targets predicted for miR-17-5p, TGFβR2 was selected for further study. TGFβR2 and its downstream TGFβ/Smad signaling were recognized as one of the vital pathway in tumor angiogenesis (Oft et al., 1998; Flores-Pérez et al., 2016; Batlle et al., 2019). Besides, previous studies have demonstrated the interaction between miR-17-5p and TGFβR2 mRNA in non-small cell lung cancers (Li et al., 2017; Chen Y. et al., 2021). Herein, we aimed to examine whether NEAT1 regulates the expression of TGFβR2 in a miR-17-5p-dependent manner. Firstly, GC cells were transfected with miR-17-5p inhibitors, mimics and their counterpart control sequences. Transfection efficiency was confirmed through RT-qPCR (Figure 3E). Furthermore, the RT-qPCR results revealed that knocking down NEAT1 in AGS and MGC803 cells significantly upregulated the expression of miR-17-5p, accompanied with downregulation of TGFβR2 expression (Figure 3F). And we investigated that the expression of miR-17-5p was negatively associated with NEAT1expression in GC tissues (Figure 3G). Additionally, the effects of miR-17-5p on TGFβR2 expression were detected by western blot and the results indicated that the expression of miR-17-5p markedly negatively correlated with TGFβR2 expression in GC cells (Figure 3H). These results collectively proved that NEAT1 positively regulated TGFβR2 expression *via* sponging miR-17-5p in gastric cancer cells.

### Repressing miR-17-5p Reverses Suppressive Effect of Silencing NEAT1 on Malignant Phenotypes of GC *in vitro*

**FIGURE 4:**
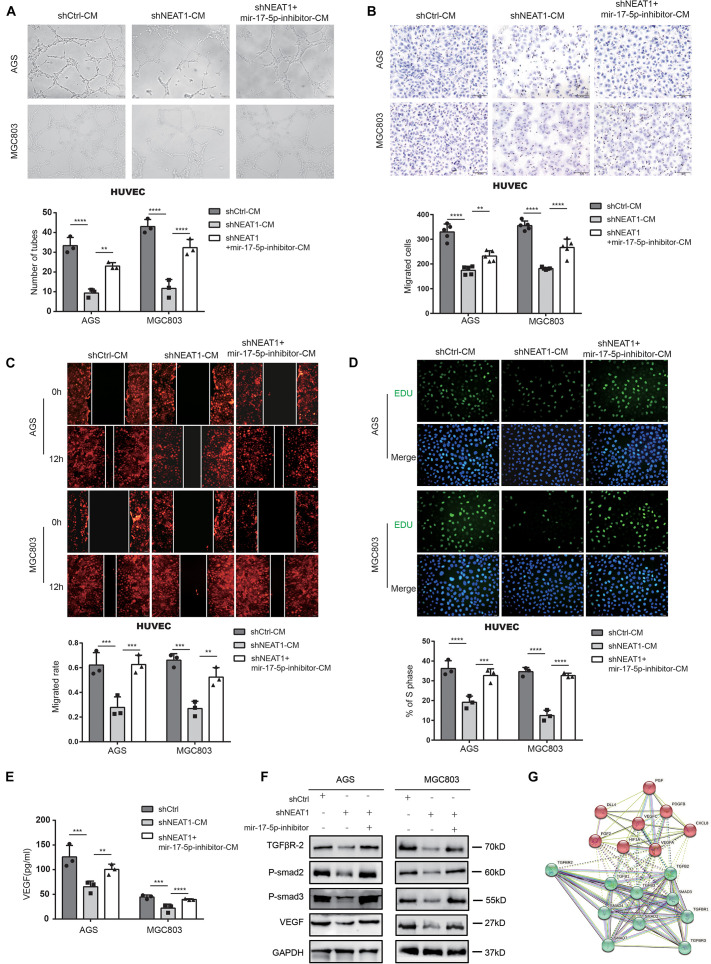
Repressing miR-17-5p reverses suppressive effect of silencing NEAT1 on malignant phenotypes of GC *in vitro.* **(A)** Effects of miR-17-5p inhibitor on NEAT1 mediated GC angiogenesis through tube formation assay. **(B,C)** Effects of miR-17-5p inhibitor on NEAT1 mediated HUVEC migration through transwell **(B)** and wound healing assay **(C)**. **(D)** Effects of miR-17-5p inhibitor on NEAT1 mediated HUVEC proliferation through EdU assay. **(E)** Effects of miR-17-5p inhibitor on secretion changes of VEGF modulated by LncRNA NEAT1 through ELISA assay. **(F)** Effects of miR-17-5p inhibitor on expression changes of TGF-β/smad pathway proteins (P-smad2, P-smad3 and VEGF) modulated by LncRNA NEAT1 through western blot assay. **(G)** The PPI network of TGF-β/smad pathway-related proteins and classsical proangiogenic factors associated with TGFβR2 alterations in String analysis. **P < 0.01, ***P < 0.001, ****P < 0.0001.

Rescue experiments were conducted using miR-17-5p inhibitors to investigate whether NEAT1 promoted angiogenesis *via* the NEAT1/miR-17-5p/TGFβR2 axis. As shown in Figure 4, miR-17-5p inhibitor dramatically reversed the inhibitory effects of NEAT1 silencing on the tube formation ability (Figure 4A), migration (Figures 4B,C) and proliferation (Figure 4D) of HUVECs. Further ELISA assay indicated that inhibiting miR-17-5p markedly reversed the inhibitory effects of NEAT1 silencing on VEGF production (Figure 4E). Additionally, we conducted western blot assay to explored the effects of NEAT1/miR-17-5p/TGFβR2 axis on the TGFβ/Smad pathway. As expected, the results revealed that silencing NEAT1 in GC cells markedly decreased expression level of TGFβR2, P-Smad2, P-Smad3, and VEGF, pivotal indicators of TGF-β/smad pathway activity. Inhibiting miR-17-5p markedly reversed the inhibitory effects of NEAT1 silencing on the aforementioned proteins (Figure 4F). We also constructed the PPI (Protein-protein interaction) network for TGFβR2, TGFβ/Smad pathway-related proteins and the aforementioned proangiogenic factors. The results indicated that these proteins were closely associated with TGFβR2 alterations (Figure 4G). In conclusion, these findings indicated that NEAT1 promotes GC angiogenesis by regulating the miR-17-5p/TGFβR2 axis.

### LncRNA NEAT1 Promotes GC Angiogenesis *via* the miR-17-5p/TGFβR2 Axis *in vivo*

**FIGURE 5:**
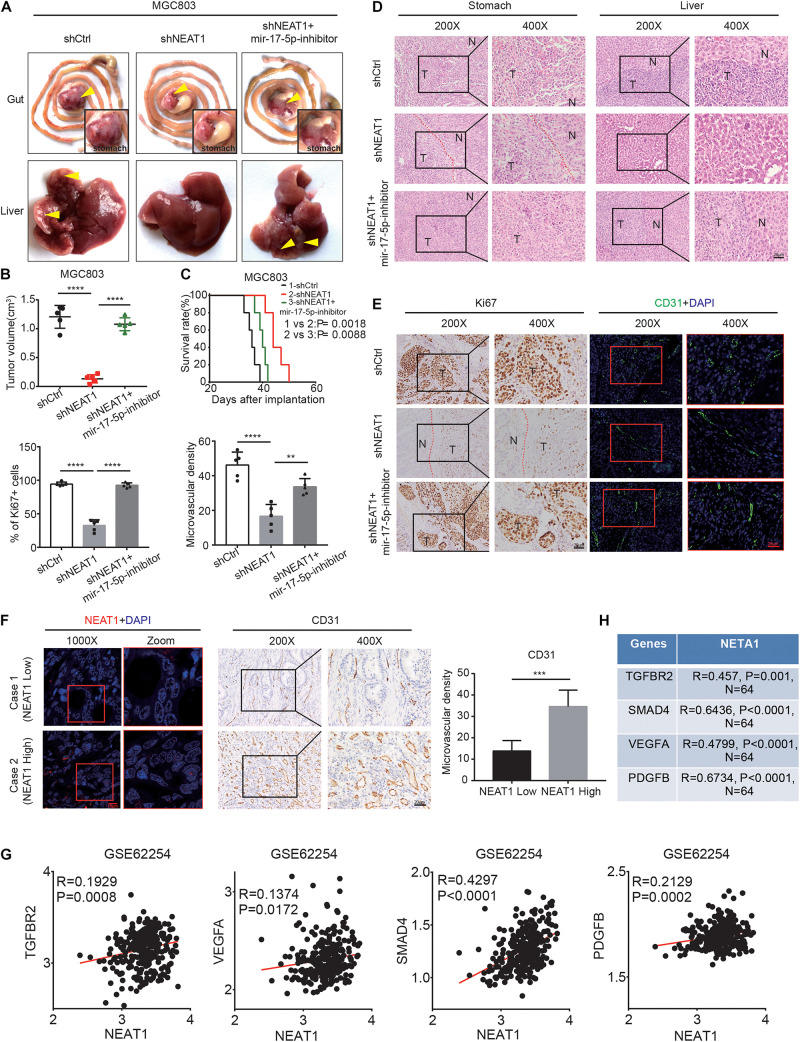
LncRNA NEAT1 promotes GC angiogenesis *via* the miR-17-5p/TGFβR2 axis *in vivo*. **(A)** Gross of GC orthotopic tumors and corresponding livers. Representative images were shown. **(B)** Size analyses of the GC orthotopic tumors. **(C)** Kaplan-Meier survival analysis of mice bearing xenografts. *n* = 5 for each group. **(D)** H&E-stained paraffin-embedded tumor obtained from xenograft tumor **(E)** IHC and IF staining for ki67 and CD31 expression in xenograft tumor. **(F)** Representative images of GC patients’ tumors with IHC and FISH staining. **(G)** Pearson correlation analysis was conducted to analyze the relation between LncRNA NEAT1, TGFβR2, VEGF, and Smad4 in GC GEO dataset. **(H)** Pearson correlation analysis was conducted to analyze the relation between LncRNA NEAT1, TGFβR2, VEGF, and Smad4 in GC tissues. **P < 0.01, ***P < 0.001, ****P < 0.0001.

To verify our *in vitro* findings, we established an *in vivo* xenograft model employing MGC803 cells in nude mice. The animal experiments were grouped as follows, shCtrl, shNEAT1, and shNEAT1 plus miR-17-5p-inhibitor. Results showed that tumor volume in the shNEAT1 group was dramatically smaller than those in the shCtrl group, and inhibiting miR-17-5p markedly reversed the inhibitory effects of NEAT1 silencing on the xenografts proliferation (Figures 5A,B). Consistent results were observed after analysing the liver metastasis rate (Supplementary Table 5) and survival time (Figure 5C) among the three groups. In addition, HE staining showed that a more regular border was observed in the shNEAT1 group than the shNC group (Figure 5D). IHC and IF assays further revealed a significant decrease in expression levels of proliferation marker Ki67 vessel marker CD31 in the shNEAT1 tumors versus the shCtrl tumors, respectively. These effects were rescued after blockade of miR-17-5p by miR-17-5p inhibitor (Figure 5E). Moreover, we collected 30 GC specimens as well as clinicopathological data. High and low NEAT1 expression were detected in GC specimens through FISH staining, respectively. As shown in Figure 5F, NEAT1 expression was positively correlated with CD31 expression (Figure 5F). Finally, a positive correlation between NEAT1 and TGFβR2, VEGF-A, Smad4, PDGFB was seen in GEO gastric cancer datasets GSE62254 (*n* = 200) (Figure 5G). The results obtained in the Nanfang group were consistent with the above results (Figure 5H and Supplementary Figure 1A). Therefore, these results further confirmed the vital role of LncRNA NEAT1/miR-17-5p/TGFβR2 signal axis in GC angiogenesis.

**FIGURE 6:**
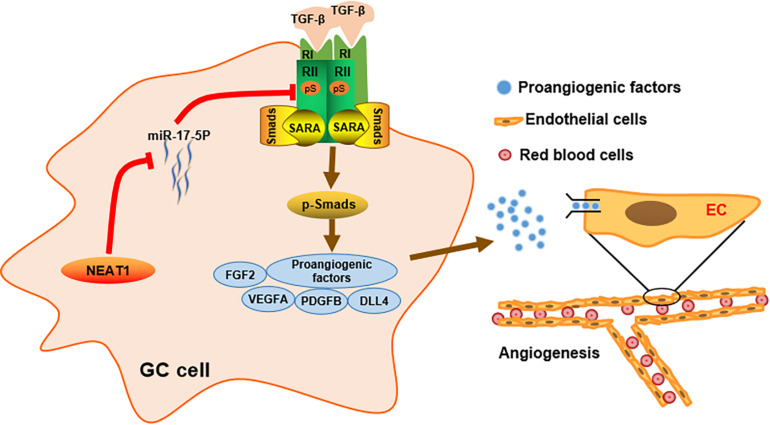
Schematic diagram of the mechanism of LncRNA NEAT1 mediated angiogenesis in gastric cancer.

## Discussion

Excessive angiogenesis is an essential characteristic of cancer, which account for the tumor proliferation (Liu et al., 2019), invasion (Zeng et al., 2018), chemoresistance (Marti et al., 2015), etc., Emerging evidences have indicated that lncRNAs participate in regulating malignant phenotypes and act as critical clinical biomarkers for diagnosis and prognosis in multiple malignancies (Yang et al., 2019; Zhao et al., 2019; Gao et al., 2020; Shi et al., 2021). Although several angiogenesis-related lncRNAs have been identified in gastric cancers (Liu H. T. et al., 2020; Teng et al., 2021), effective anti-angiogenesis strategies in GC are still limited and the underlying mechanisms remain to be further disclosed.

Our study firstly identified lncRNA NEAT1 as a novel angiogenesis regulator in GC angiogenesis. NEAT1 was markedly upregulated in GC specimens and a higher NEAT1 expression was correlated with advanced stages and poor outcome in GC patients. Furthermore, functional experiments indicated that NEAT1 disruption inhibited angiogenesis both *in vitro* and *in vivo*. Numerous studies have demonstrated that lncRNAs regulate cellular processes in cancer cells by sponging specific miRNA as a ceRNA. For instance, lncRNA LINC00657 inhibites cervical cancer progression *via* sponging miR-20a-5p and targeting RUNX3 (Qin et al., 2021). LncRAN LINC01137 interacts with miR-22-3p to promote cell proliferation and invasion in oral squamous cell carcinoma (Du et al., 2021). Consistently, through the dual luciferase reporter assay and RNA pull-down assay, we found that the oncogenic function of NEAT1 in gastric cancer was modulated through its interaction with miR-17-5p, which has been identified as an oncogenic miRNA in a variety of tumor types. Silencing NEAT1 upregulated the expression level of miR-17-5p and in turn, inhibition of miR-17-5p reversed the inhibitory effects of NEAT1 silencing on GC angiogenesis both *in vitro* and *in vivo*.

Previous studies have revealed that miR-17-5p, a member of the miR-17-92 cluster, participates in comprehensive biological processes of cancer cells by regulating expression of its target mRNA at post-transcriptional level. For example, miR-17-5p directly targets RUNX3 and promotes proliferation and invasiveness in gastric cancer (Song et al., 2020). MiR-17-5p directly targets ERBB3 and suppresses postoperative metastasis of hepatocellular carcinoma (Liu D. L. et al., 2020). Herein, we identified TGFβR2 as a direct target of miR-17-5p in GC, which was consistent with previous studies (Li et al., 2017; Chen Y. et al., 2021). TGFβR2 protein expression was significantly negatively correlated with miR-17-5p in GC cell lines. Importantly, we further elucidated the involvement of LncRNA NEAT1 in the regulatory role of miR-17-5p in TGFβR2 expression. LncRNA NEAT1 sponges miR-17-5p and decreases its expression level, which subsequently activated the TGFβR2 expression and its downstream TGFβ/Smad pathway.

Transforming growth factor-β receptor 2 is located on the cell membrane and initiates the signal transduction by interacting with the TGFβ ligands (TGFβ-1, TGFβ-2, TGFβ-3). TGFβR2 phosphorylates the kinase domain of TGFβR1, which subsequently propagates the signal by phosphorylating Smad2 and Smad3. Phosphorylated- Smad2 and Smad3 polymerize with Smad4 to form the active transcriptome complex which translocate to nucleus and regulate extensive gene expression in both biological and pathological processes (Tsukazaki et al., 1998; De Caestecker et al., 2000). Activation of the TGFβ/Smad pathway have been recognized to upregulate numerous proangiogenic factors such as VEGF (Chruścik et al., 2018), FGF-2 (Yang et al., 2008), and TSP-4 (Muppala et al., 2017) in cancers. Among the aforementioned factors, VEGF was recognized to be the dominant angiogenesis regulator based on extensive researches (Leung et al., 1989; Carmeliet et al., 1996; Ferrara et al., 1996; Chen Z. et al., 2021). Herein, we demonstrated that LncRNA NEAT1 regulated TGFβR2 expression in a miR-17-5p-dependent manner. Silencing NEAT1 inhibited TGFβR2 expression as well as its downstream TGFβ signaling, accompanied with downregulation of a series of proangiogeneic factors especially VEGF. Our results are consistent with previous studies. However, further *in vitro* and *in vivo* experiments are required to validate the effects of TGFβR2 on LncRNA NEAT1-mediated angiogenesis in GC.

## Conclusion

In conclusion, our study identified lncRNA NEAT1 as a novel driving factor in GC angiogenesis. We demonstrated *in vitro* and *in vivo* that lncRNA NEAT1 promoted TGFβR2 expression *via* competitively sponging miR-17-5p, thereby activated the TGFβ/Smad pathway and upregulated expression of proangiogenic factors especially VEGF. These findings suggest that the lncRNA NEAT1/miR-17-5p/TGFβR2 signal axis may provide promising targeting strategies for GC diagnosis and treatment.

## Data Availability Statement

The raw data supporting the conclusions of this article will be made available by the authors, without undue reservation.

## Ethics Statement

The studies involving human participants were reviewed and approved by Ethical Committee of Nanfang Hospital. The patients/participants provided their written informed consent to participate in this study. The animal study was reviewed and approved by Animal Care and Use Committee of Southern Medical University.

## Author Contributions

QZ led the study design and supervised this work. YX prepared the manuscript and analyzed the data. YX, YL, YQ, FS, GZ, JS, GC, WL, YF, and HW performed the experiments. SJ, ZW, FF, JL, and YY gave assistance in collecting tissue samples and animal experiments. All authors discussed the results and approved of the final version.

## Conflict of Interest

The authors declare that the research was conducted in the absence of any commercial or financial relationships that could be construed as a potential conflict of interest.

## Publisher’s Note

All claims expressed in this article are solely those of the authors and do not necessarily represent those of their affiliated organizations, or those of the publisher, the editors and the reviewers. Any product that may be evaluated in this article, or claim that may be made by its manufacturer, is not guaranteed or endorsed by the publisher.

## Footnotes

1
http://kmplot.com/analysis/index.php?p=service
