# Supplementary figures and images for: LncRNA NEAT1 Promotes Gastric Cancer Progression Through miR-17-5p/TGFβR2 Axis Up-Regulated Angiogenesis
Source: Front Cell Dev Biol. 2021 Sep 6;9:705697. doi: 10.3389/fcell.2021.705697 (PMC8452045; doi:10.3389/fcell.2021.705697)

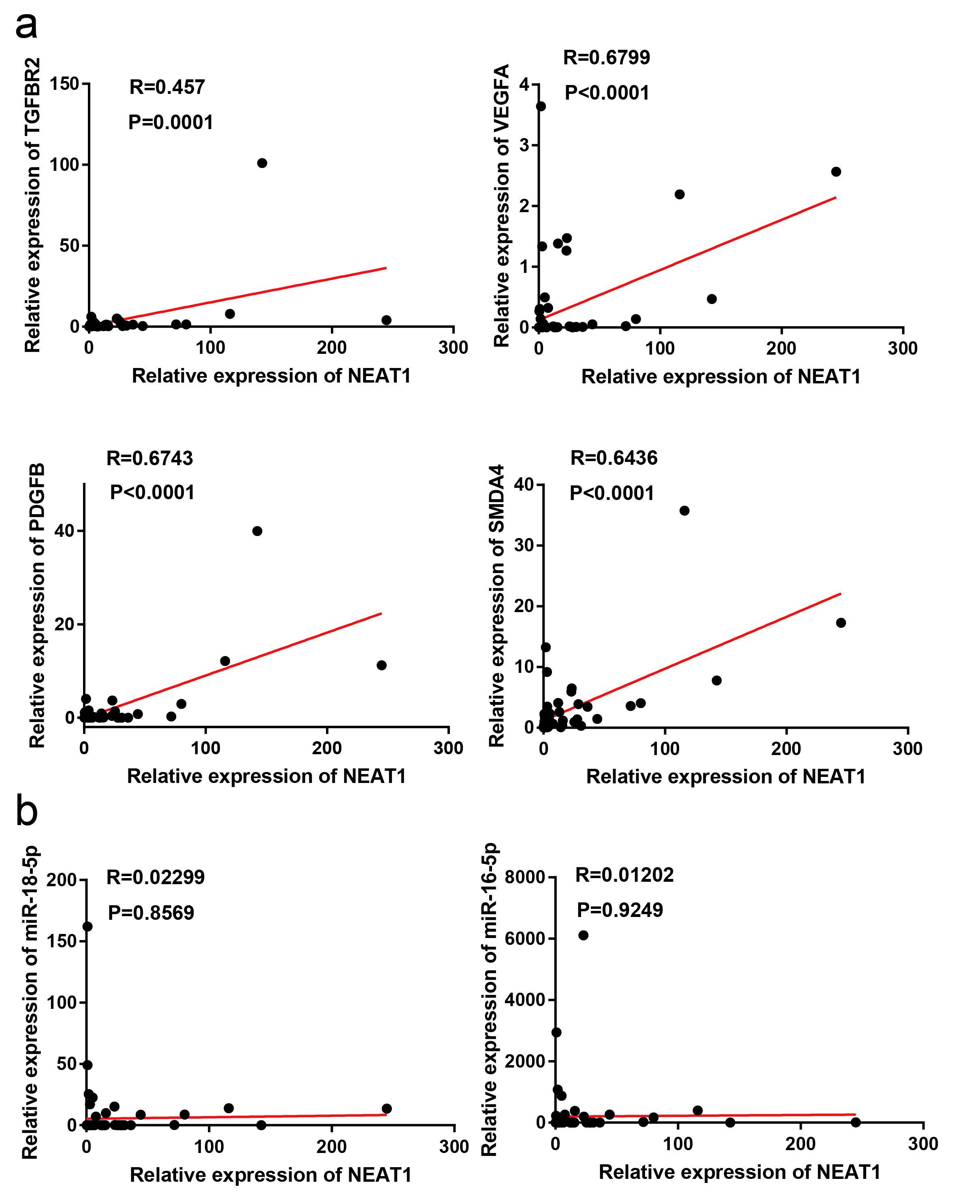

Supplement: Supplementary file 1 [file Image_1.TIF]

# NEAT1 (214657\_s\_at)

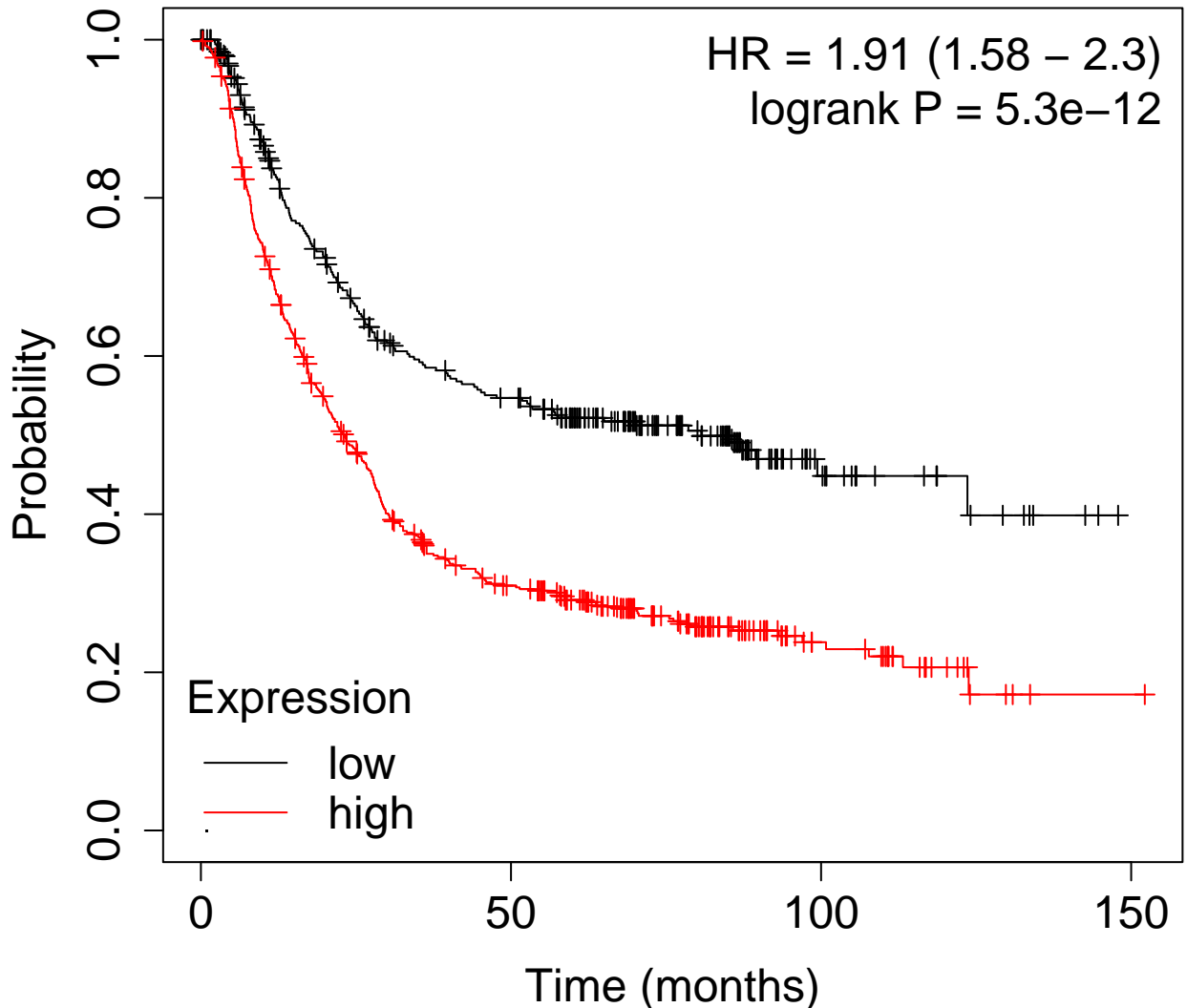

Number at risk

|      |     |     |    |   |
|------|-----|-----|----|---|
| low  | 343 | 156 | 21 | 0 |
| high | 532 | 142 | 27 | 1 |

Supplement: Supplementary file 3 [file Data_Sheet_1.ZIP › figure 1-raw data/figure 1e.pdf]

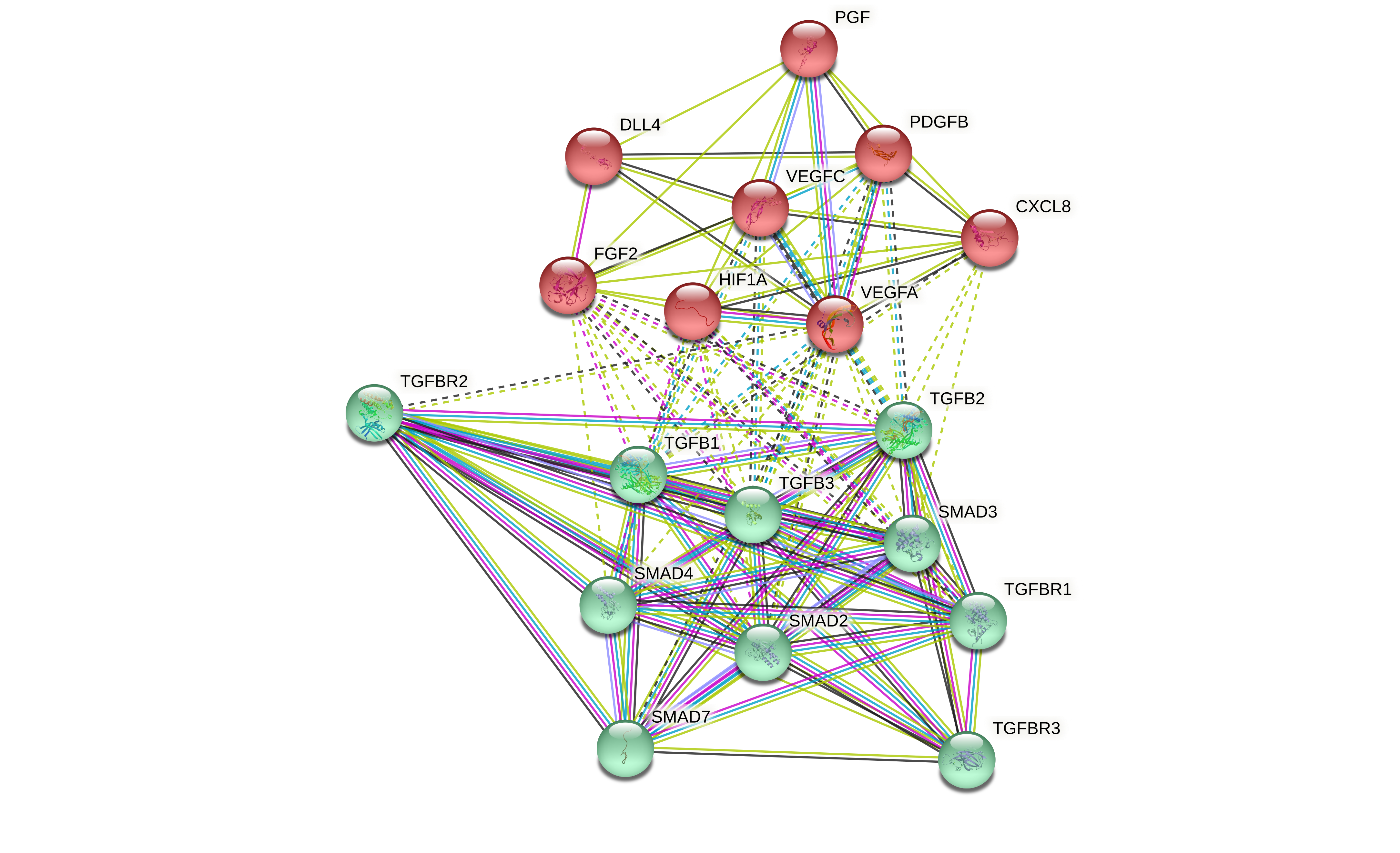

Supplement: Supplementary file 6 [file Data_Sheet_4.ZIP › figure 4-raw data/figure 4g.png]
