# Supplementary material for: LncRNA NEAT1 Promotes Gastric Cancer Progression Through miR-17-5p/TGFβR2 Axis Up-Regulated Angiogenesis
Source: Front Cell Dev Biol. 2021 Sep 6;9:705697. doi: 10.3389/fcell.2021.705697 (PMC8452045; doi:10.3389/fcell.2021.705697)
Supplement: Supplementary file 2 [file Table_1.DOCX]

**Supplementary Table S1** Correlations between the expression of LncRNA NEAT1 and various clinicopathological features in 64 GC patients

| Parameters | Characteristic | NEAT1 expression | | P value |
| --- | --- | --- | --- | --- |
|  |  | High (*n*=51) | Low (*n*=13) |  |
| Age (years) | ≥60 | 26 | 2 | **0.0209*** |
|  | <60 | 25 | 11 |  |
| Gender | Male | 34 | 8 | 0.7282 |
|  | Female | 17 | 5 |  |
| Tumor size (cm) | ≥5 | 30 | 1 | **0.001**** |
|  | <5 | 21 | 12 |  |
| T stage | T1-T2 | 9 | 8 | **0.0014**** |
|  | T3-T4 | 42 | 5 |  |
| N stage | N0-N1 | 31 | 10 | 0.279 |
|  | N2-N3 | 20 | 3 |  |

(*:p<0.05; **:p<0.01)

**Supplementary Table S2.** RT-qPCR primer sequences for human genes

| **Gene** | **Forward primer** | **Reverse primer** | **Product length** |
| --- | --- | --- | --- |
| GAPDH | GGAGCGAGATCCCTCCAAAAT | GGCTGTTGTCATACTTCTCATGG | 197bp |
| NEAT1 | GGCCAGAGCTTTGTTGCTTC | GGTGCGGGCACTTACTTACT | 91bp |
| FGF2 | TTCACAGCCCTGACCGAGAA | CGTTGCTACAGTAGAGGAGTTTG | 76bp |
| VEGFA | AGGGCAGAATCATCACGAAGT | AGGGTCTCGATTGGATGGCA | 75bp |
| VEGFC | GAGGAGCAGTTACGGTCTGTG | TCCTTTCCTTAGCTGACACTTGT | 96bp |
| PDGFB | CTCGATCCGCTCCTTTGATGA | CGTTGGTGCGGTCTATGAG | 239bp |
| CXCL8 | TTTTGCCAAGGAGTGCTAAAGA | AACCCTCTGCACCCAGTTTTC | 194bp |
| DLL4 | GTCTCCACGCCGGTATTGG | CAGGTGAAATTGAAGGGCAGT | 98bp |
| HIF1A | GAACGTCGAAAAGAAAAGTCTCG | CCTTATCAAGATGCGAACTCACA | 124bp |
| TGF-β1 | CGCTGCCCATCGTGTACTA | ACACAGAGATCCGCAGTCCT | 240bp |
| PGF | GAACGGCTCGTCAGAGGTG | ACAGTGCAGATTCTCATCGCC | 187bp |

**Supplementary Table S3.** RT-qPCR primer sequences for mircoRNA

| **List of oligonucleotide**  **sequences** | **5’→3’** |
| --- | --- |
| **Primers for Real-time PCR and RT-PCR** | |
| U6—qF | CTCGCTTCGGCAGCACA |
| U6—qR | AACGCTTCACGAATTTGCGT |
| hsa-miR-16-5p | CCCGTAGCAGCACGTAAATA |
| hsa-miR-301a-3p | CCCTAGTATTGTCAAAGCAAAA |
| hsa-miR-93-5p | CCCAAAGTGCTGTTCGTGC |
| hsa-miR-20a-5p | TAAAGTGCTTATAGTGCAGGTA |
| hsa-miR-17-5p | CAAAGTGCTTACAGTGCAGGT |
| hsa-miR-27a-3p | TTCACAGTGGCTAAGTTCCG |
| hsa-miR-15a-5p | TAGCAGCACATAATGGTTTGT |
| hsa-mir-27b-3p | TTCACAGTGGCTAAGTTCTGC |
| hsa-miR-20b-5p | AAGTGCTCATAGTGCAGGTAG |
| hsa-miR-18a-5p | UAAGGUGCAUCUAGUGCAGAUA |
| hsa-miR-374a-5p | GTTATAATACAACCTGATAAGTG |

**Supplementary Table S4.** Ten miRNAs were identified as the potential targets of NEAT1

| **miRNAid** | **miRNAname** | **geneID** | **start** | **end** | **miRseq** | **targetSeq** |
| --- | --- | --- | --- | --- | --- | --- |
| MIMAT0000072 | hsa-miR-18a-5p | ENSG00000245532 | 65206598 | 65206622 | gauagAC-GUGAU-CUACGUGGAAu | augagUGAUGCUAUCAAGCACCUUu |
| MIMAT0000688 | hsa-miR-301a-3p | ENSG00000245532 | 65204086 | 65204109 | cgaaacUGUUAUG-AUAACGUGAc | caccacACAGCCUGUCUUGCACUg |
| MIMAT0000068 | hsa-miR-15a-5p | ENSG00000245532 | 65209688 | 65209710 | guguuUGGUAAUAC-ACGACGAu | gccucGCCUUCACGCUGCUGCUg |
| MIMAT0000070 | hsa-miR-17-5p | ENSG00000245532 | 65210090 | 65210111 | gaUGGACGUGACAUUCGUGAAAc | acGCCUG-AAUCUUAGCACUUUg |
| MIMAT0000084 | hsa-miR-27a-3p | ENSG00000245532 | 65192982 | 65193001 | cgccUUGAAUCGGUGACACUu | gguuGAUUU-GAAACUGUGAa |
| MIMAT0000419 | hsa-miR-27b-3p | ENSG00000245532 | 65192982 | 65193001 | cgucUUGAAUCGGUGACACUu | gguuGAUUU-GAAACUGUGAa |
| MIMAT0000103 | hsa-miR-106a-5p | ENSG00000245532 | 65206890 | 65206912 | gaUGGACGUGACAUUCGUGAAAa | acGCUUGUAAUCCCAGCACUUUg |
| MIMAT0000093 | hsa-miR-93-5p | ENSG00000245532 | 65206890 | 65206912 | gaUGGACGUGCUUGUCGUGAAAc | acGCUUGUAAUCCCAGCACUUUg |
| MIMAT0000727 | hsa-miR-374a-5p | ENSG00000245532 | 65199856 | 65199877 | gugaauaguccaacAUAAUAUu | gugcaaaaagcaccUAUUAUAc |
| MIMAT0000069 | hsa-miR-16-5p | ENSG00000245532 | 65209688 | 65209710 | gcgguuauAAAUGC-ACGACGAu | gccucgccUUCACGCUGCUGCUg |

**Supplementary Table S5: NEAT1 and NEAT1/** **miR-17-5p modified orthotopic xenograph GC tumor formation and liver metastasis analysis.**

|  | Total mice  number | Orthotropic tumor N(%) | High tumor burden N(%) | | Liver metastasis  N(%) | |
| --- | --- | --- | --- | --- | --- | --- |
| MGC803.shCtrl  MGC803.shNEAT1  MGC803.shNEAT1  miR-17-5p-inhibitor | 5  5  5 | 5(100）  5(100)  5(100) | 5(100)  1(20)  5(100) | 2(40)  0(0)  1(20) | |  |

High tumor burden: The tumor mass is more than 1cm in diameter
